# Commentary: Novel Cell Culture Paradigm Prolongs Mouse Corneal Epithelial Cell Proliferative Activity *In Vitro* and *In Vivo*

**DOI:** 10.3389/fcell.2022.822728

**Published:** 2022-02-18

**Authors:** Xiuna Ji, Mingyue Zheng, Tingjun Fan, Bin Xu

**Affiliations:** ^1^ College of Marine Life Sciences, Ocean University of China, Qingdao, China; ^2^ Guangdong Provincial People’s Hospital, Guangdong Academy of Medical Sciences, Guangzhou, China

**Keywords:** limbal stem cells, self-renewal, *in vitro* expansion, small-molecule compounds, regenerative medicine

## 1 Introduction

Limbal stem cells (LSCs) are located in the limbal palisades of Vogt between the cornea and conjunctiva. The LSCs exhibit a series of key characteristics of epithelial stem cells, including self-renewal ability, high proliferation potential, and tissue regeneration capacity (Li et al., 2021a). The loss of LSCs and damage to their microenvironment contribute to various corneal diseases such as LSC deficiency (LSCD). LSCD further causes corneal vascularization, opacification, and even blindness (Singh and Sangwan, 2021). Better treatments for LSCD are currently being developed, including LSC culture for autograft. In 2015, Holoclar [*ex vivo* expanded autologous human corneal epithelial cells (CECs) containing stem cells] successfully gained the marketing authorization from the European Medicines Agency to treat moderate-to-severe LSCD following chemical and thermal eye burns (Yu et al., 2018). The regenerative potential of Holoclar mainly relies upon the highly proliferative and self-renewing properties of holoclones. However, it still needs to be further explored on how to optimize LSC expansion *in vitro*. In fact, current efforts are focused on the addition of small molecular compounds to improve efficiency of stem cell expansion. These compounds have clear targets, show quick action and reversibility, and are therefore of great value in the research and practical application toward maintaining LSC self-renewal.

## 2 A Novel Strategy to Optimize Self-Renewal and Expansion of Limbal Stem Cells in Culture

It is well known that increasing extracellular calcium, serum and air-lifting can lead to terminal differentiation and gradual loss of LSCs (Meyer-Blazejewska et al., 2010). Previously, Xiang et al. has reported the long-term *in vitro* maintenance of primary human hepatocytes by modulating cell signaling pathways with a combination of five chemicals (5C), including forskolin, SB431542, DAPT, IWP-2, and LDN-193189 (Xiang et al., 2019). Interestingly, each compound of 5C has been proved to improve corneal epithelium homeostasis. This study by An et al. (2021) compared the effects of 5C and 6C (combined Y-27632 and 5C) on the mouse CECs (mCECs) and found that 6C could increase mCEC proliferation, sustain the expression levels of the progenitor cell function gene, as well as suppress epithelial–mesenchymal transition. The 6C culture method may be applied for improving the availability of CECs to treat LSCD in clinical practice.

The 6C improve the maintenance of mCEC morphology and function in long-term culture, subculture *in vitro*, and mouse cornea culture *ex vivo*. Moreover, the 6C culture system conduces to construct tissue-engineered corneal epithelium and promotes healing of corneal epithelial wounds in mice. These small-molecule combinations regulate mCEC proliferation involving in cAMP, TGF-β, BMP, Notch, Wnt/β-catenin, and Rho/ROCK signaling (An et al., 2021). Remarkably, 6C seem to maintain the limbal proliferating stem and progenitor cell phenotype *in vivo*, which demonstrates that these signaling modulators might regulate LSC functions. Considering that CECs have a finite capacity to replicate and eventually enter irreversible growth arrest, it is more significant to apply this novel strategy to LSC expansion based on the mechanisms underlying LSC self-renewal. Then, specific small-molecule compounds can be optimized to act on the signaling regulatory network for LSC expansion in culture.

Transcription factor (TF) PAX6 is expressed during eye development, which is considered as the master gene for oculogenesis (Ramos et al., 2015). PAX6 plays an essential role in specifying LSCs, in which Wnt7a controls CEC fate determination through PAX6 (Ouyang et al., 2014). GSK3β inhibitors, lithium chloride, and CHIR-99021 can activate the canonical Wnt pathway to improve LSC self-renewal (Bonnet et al., 2021). Specific small molecules such as IIIC3 (DKK inhibitor) and MFH-ND (Wnt mimic) have been designed to improve LSC expansion *in vitro* by interacting specifically with the Wnt co-receptors LRP5/6 and FZD (Janda et al., 2017; Gonzalez et al., 2019; Chen et al., 2020; Zhang et al., 2020). TFs RUNX1 and SMAD3 are also required for maintenance of corneal epithelial identity and homeostasis by interactions with PAX6 (Li et al., 2021c). Moreover, RUNX1 can shape LSC chromatin architecture *via* modulating H3K27ac deposition (Li et al., 2021c). RNA sequencing (RNA-seq) and qualitative proteomics identify that miR146a has an opposite regulatory role in the fine-tuning of Notch 1/2 expression to balance LSC self-renewal and differentiation (Poe et al., 2020). The above studies suggest that LSC self-renewal is regulated by a variety of signaling pathways ranging from signaling factor, TF, epigenetic regulator to microRNA, which serve as putative targets of small-molecule compounds during LSC culture.

LSCs certainly need to communicate with their own niche to maintain self-renewal (Li et al., 2007), including specific extracellular matrix (ECM), niche cells, and signaling molecules (Ashworth et al., 2021). The ECM not only anchors the basal epithelium but also mediates intercellular communication and provides distinct mechanical properties that influence LSC phenotype, population, and self-renewal (Gesteira et al., 2017; Gouveia et al., 2019; Zheng et al., 2019; Zhu et al., 2020; Ashworth et al., 2021). Additionally, cell–cell communication analysis reveals the central role of LSCs and their bidirectional regulation with various niche cells, such as limbal–mesenchymal stem cells, CD45^+^ cells, PAX6^+^ cells, and melanocytes (Yazdanpanah et al., 2019; Altshuler et al., 2021; Chen et al., 2021; Polisetti et al., 2021). The surrounding niche cells regulate LSC homeostasis by modulating signaling pathways such as Wnt, Notch, TGF-β, and BMP signaling (Bonnet et al., 2021). These indicate that LSC culture surface needs to satisfy the component and stiffness of limbal niche to regulate the signaling network of LSC self-renewal which might be used as effective targets of specific small molecules. Significantly, it is now well-accepted that culturing cells in three-dimensional systems that mimic key factors of tissues is much more representative of the *in vivo* microenvironment than simple two-dimensional monolayers (Langhans, 2018). Therefore, the small-molecule application shows its advantage for LSC expansion in a niche-mimicking culture system *via* cellular signaling crosstalk.

Single-cell RNA-seq (scRNA-seq) technologies are broadly applied to dissect the cellular heterogeneity and expression dynamics, providing unprecedented insights into single-cell biology (Plass et al., 2018; Li et al., 2020). A few groups have recently used scRNA-seq to analyze LSC populations and corneal homeostasis (Li et al., 2021a; Li et al., 2021b). These data provide a new avenue for studying the mechanisms of LSC self-renewal (Altshuler et al., 2021). Single-cell transcriptomics have identified that TFs (TP63, MYC, PARP1, and SOX17) have important roles in regulating LSC stemness and proliferation (Li et al., 2021a; Dou et al., 2021). Moreover, two novel surface markers (GPHA2 and TSPAN7) support LSC self-renewal (Li et al., 2021a; Collin et al., 2021), suggesting that GPHA2^+^ and/or TSPAN7^+^ LSCs can be isolated from multiple cell types in limbus by flow cytometry and cell sorting technology. Significantly,, thioredoxin-interacting protein (TXNIP), a metabolic protein involved in redox regulation, is highly expressed in LSCs, which may be a novel LSC-preferred gene that contributes to stem cell maintenance (Kaplan et al., 2019). It also indicates that there might be a close relationship between cellular metabolism and LSC self-renewal. The combination of single-cell transcriptomics and metabolomics appears to help us comprehensively understand the mechanisms of LSC self-renewal. And these multi-omics analysis will provide inspiring evidence to develop small-molecule compounds for LSC expansion.

## 3 Conclusion

**FIGURE 1:**
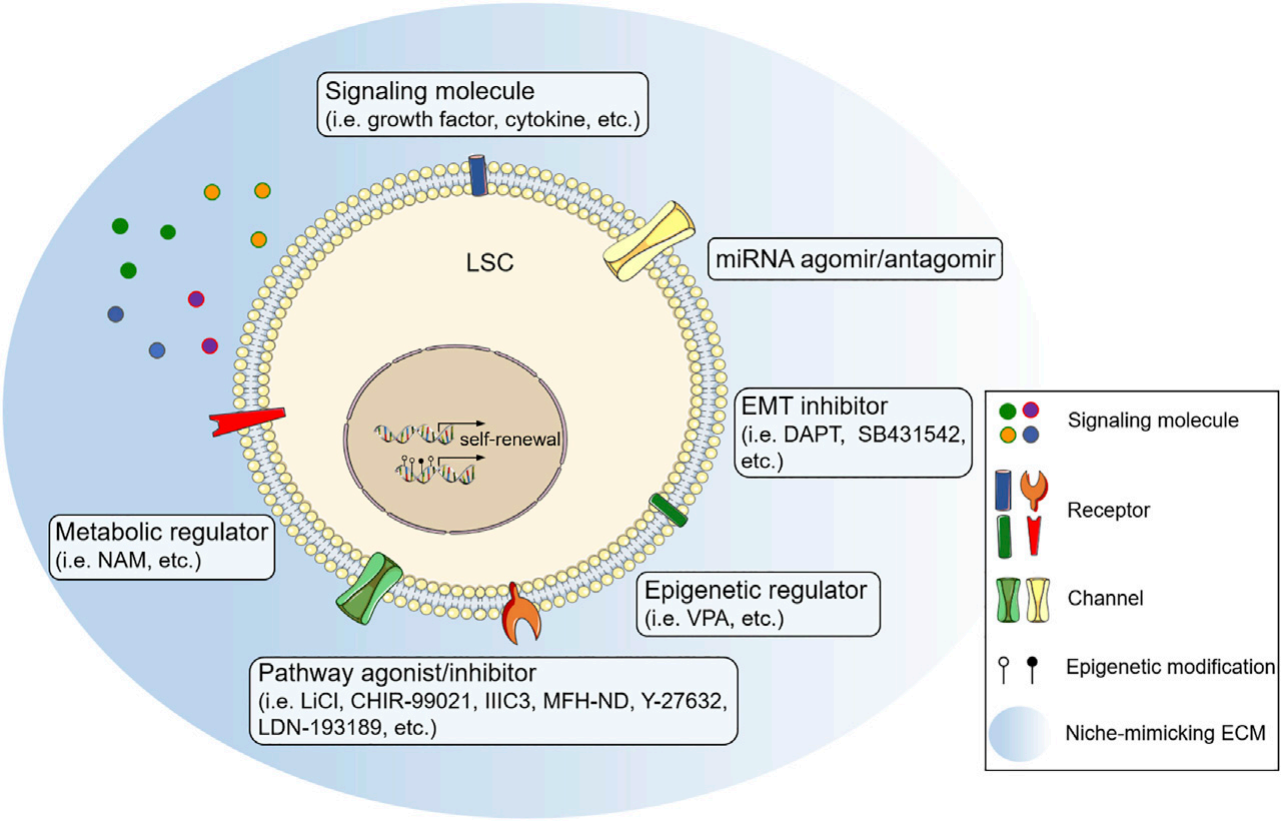
Small-molecular combination as a novel strategy to optimize limbal stem cell (LSC) expansion in culture. GSK3β inhibitors [lithium chloride (LiCl), CHIR-99021], DKK inhibitor (IIIC3), and Wnt mimic (MFH-ND) activate the canonical Wnt pathway to improve LSC expansion. Growth factor (pigment epithelial-derived factor), cytokine (leukemia inhibitory factor), ROCK inhibitor (Y-27632), and BMP signaling inhibitor (LDN-193189) also promote LSC self-renewal through multiple signaling pathways. TGF/β signaling inhibitor (SB431542) and Notch signaling inhibitor (DAPT) prevent epithelial–mesenchymal transition of LSC by inhibiting the corresponding pathways. Histone deacetylase inhibitor (VPA), miRNA agomir/antagomir and nicotinamide adenine dinucleotide (NAD^+^) precursor [nicotinamide (NAM)] might enhance the efficiency of LSC expansion through the regulation at epigenetic, post-transcriptional, and metabolic levels. ECM, extracellular matrix; EMT, epithelial-mesenchymal transition; miRNA, microRNA.

The long-term maintenance of cell function requires a sophisticated signaling regulatory network; a chemical strategy using small-molecule combinations confers the advantage of synergistically orchestrating innate signals to achieve spatiotemporal modulations of specific cellular targets (Xu et al., 2015). This is the first report on the 6C culture system prolonging mCEC maintenance *in vitro*. The chemical approach is simple and easily applied for autologous epithelial sheet transplantation. It also provides a new idea and method for LSC expansion *in vitro* (Figure 1). More stable LSC populations can be obtained for applications in regenerative medicine research by optimizing specific small-molecule combinations under a niche-mimicking culture condition.
